# Effect of hydrocephalus on rat brain extracellular compartment

**DOI:** 10.1186/1743-8454-5-12

**Published:** 2008-07-10

**Authors:** Marc R Del Bigio, Terry L Enno

**Affiliations:** 1 Department of Pathology, University of Manitoba, and Manitoba Institute of Child Health, Winnipeg MB, R3E 3P5, Canada

## Abstract

**Background:**

The cerebral cortex may be compressed in hydrocephalus and some experiments suggest that movement of extracellular substances through the cortex is impaired. We hypothesized that the extracellular compartment is reduced in size and that the composition of the extracellular compartment changes in rat brains with kaolin-induced hydrocephalus.

**Methods:**

We studied neonatal (newborn) onset hydrocephalus for 1 or 3 weeks, juvenile (3 weeks) onset hydrocephalus for 3–4 weeks or 9 months, and young adult (10 weeks) onset hydrocephalus for 2 weeks, after kaolin injection. Freeze substitution electron microscopy was used to measure the size of the extracellular compartment. Western blotting and immunohistochemistry with quantitative image densitometry was used to study the extracellular matrix constituents, phosphacan, neurocan, NG2, decorin, biglycan, and laminin.

**Results:**

The extracellular space in cortical layer 1 was reduced significantly from 16.5 to 9.6% in adult rats with 2 weeks duration hydrocephalus. Western blot and immunohistochemistry showed that neurocan increased only in the periventricular white matter following neonatal induction and 3 weeks duration hydrocephalus. The same rats showed mild decorin increases in white matter and around cortical neurons. Juvenile and adult onset hydrocephalus was associated with no significant changes.

**Conclusion:**

We conclude that compositional changes in the extracellular compartment are negligible in cerebral cortex of hydrocephalic rats at various ages. Therefore, the functional change related to extracellular fluid flow should be reversible.

## Background

Hydrocephalus is characterized by enlargement of the cerebrospinal fluid (CSF)-containing ventricles of the brain. Several factors including tissue compression, stretching of axons, ischemia, and calcium-mediated proteolysis simultaneously contribute to axon injury in periventricular white matter [1,2]. We have also postulated that movement and composition of extracellular fluids are altered with potential reversible effects on neuronal function [3] and Hakim suggested that the brain behaves like a compressed sponge with reduction of the extracellular compartment [4]. Although the periventricular white matter extracellular compartment is enlarged in progressive hydrocephalus [3,5], several types of evidence suggest that the more superficial parts of brain are indeed compressed. Tissue density measurements in adult rabbits with silicone oil-induced hydrocephalus indicated increased density (possibly decreased water content) in cortical gray matter [6], although this was not replicated in a similar study that used wet-dry weight comparisons [7]. Computed tomography scanning of human brains suggests that brain hydration is reduced when the ventricles expand [8]. Freeze-substitution electron microscopic studies of hy-3 mutant mice show that the extracellular compartment in the superficial cerebral cortex is initially compressed and subsequently enlarged [9]. Increased electrical impedance has been interpreted as a reflection of decreased water content in thalamus of hydrocephalic cats [10]; similar findings have been obtained in human studies [11,12]. Apparent diffusion coefficient (ADC) mapping in rats with hydrocephalus suggest that the movement of water in rat brain is restricted in gray matter but not in white matter [13]. Extracellular infusion of gadolinium-DTPA followed by repeated magnetic resonance (MR) imaging [14] and real-time iontophoretic tetramethylammonium diffusion studies [15] suggest that the extracellular flow and volume fraction is reduced in the cerebral cortex of rats with kaolin-induced hydrocephalus. In the H-Tx rat with congenital hydrocephalus, ionic composition studies suggest that the extracellular compartment in the cortex is enlarged 4–21 days after birth [16] while iontophoretic studies indicate that the extracellular volume fraction is reduced [15]. In conjunction with the obstruction to CSF outflow, composition of the CSF is altered [17].

It is important to determine if this impairment of extracellular flow in hydrocephalic brains is purely due to physical compression, which could be reversible upon shunting, or if there are structural or compositional changes in the extracellular compartment. If the latter is true, then flow of metabolic waste products might not be reversible by shunting. We hypothesized that hydrocephalus is associated with narrowing of the extracellular space and that chronic hydrocephalus is associated with changes in the extracellular matrix composition. The goals of this experiment were to define the compositional changes of rat brain extracellular matrix constituents in neonatal acute and subacute, juvenile subacute and chronic hydrocephalus rats in comparison to age-matched controls, and to examine anatomical and functional changes in the extracellular compartment. We studied the chondroitin/dermatan sulfate proteoglycans: phosphacan [18], neurocan (which is a member of the lectican family) [19], and NG2 [20], and the two small leucine-rich proteoglycans: decorin and biglycan [21]. These extracellular proteins have chains of glycosaminoglycans bound to core proteins. With the additional use of less specific histochemical stains, we covered most of the major classes of extracellular substances in the brain. We also studied the basal lamina-associated protein laminin [22]. We used freeze-substitution electron microscopy [23,24] to assess relative dimensions of the cortical extracellular space. This method is one of the only ways to assess the physical characteristics of the extracellular compartment independent of its functional status by avoiding the cellular swelling that occurs using conventional fixation methods [25,26]. It is, however, limited in the depths to which preservation is acceptable.

## Methods

### Induction and assessment of hydrocephalus

All animals were treated in accordance with guidelines set forth by the Canadian Council on Animal Care and the institutional animal ethics committee approved protocols. Locally bred Sprague Dawley rats (n = 40) were used for the biochemical and histological experiments. Hydrocephalus was induced by percutaneous injection of a sterile suspension of kaolin (250 mg/ml in 0.9% saline) through a 28-gauge needle into the cisterna magna. Newborn pups were immobilized by cold anesthesia, injected with kaolin (0.02 ml), and allowed to survive 1 or 3 weeks, after which neurological impairments became too severe. Three-week rats and young adults (10 weeks) were anesthetized by intramuscular ketamine/xylazine (90/5 mg/kg), the neck was shaved and flexed, and under aseptic conditions kaolin was injected (0.04 ml). Three-week rats were allowed to survive 4 weeks or 9 months. Young adults were allowed to survive 2 weeks; they developed impairment related to raised intracranial pressure with only mild ventricular enlargement. Table 1 summarizes these groups. In all of the text, the age of the rats refers to the age at time of sacrifice. Control animals received a sham injection with needle insertion only. Animals were housed with their mothers or after weaning 2–3 per standard cage with normal 12-h day/night lighting and water and food *ad libitum*. Moistened food and water were provided on the floor of the cages for neurologically impaired animals.

**Table 1:** Hydrocephalic rat groups studied (along with age-matched controls)

Age at kaolin injection	Duration of hydrocephalus	Age at sacrifice
1 day	1 week	1 week
1 day	3 weeks	3 weeks
3 weeks	3–4 weeks	7 weeks
10 weeks	2 weeks	12 weeks
3 weeks	9 months	10 months

In the text we refer to the age at time of sacrifice

MR imaging was performed on all rats within 24 hours of sacrifice using a Bruker Biospec/3 MR scanner equipped with a 21-cm bore magnet operating at a field of 7T (Karlsruhe, Germany) to obtain T2-weighted images of the brain in the coronal plane. The area of the lateral ventricles and cerebrum were measured in the rostral cerebrum slice (0.5 mm thickness) immediately anterior to the third ventricle. Only rats with significant ventriculomegaly (ventricle to cerebrum area ratio >0.25, except for the rats injected at 10 weeks, which never develop severe ventricular enlargement, ventricle to cerebrum area ratio >0.10; control value <0.01) were used in this study along with sham-injected controls (n = 4–5 for each age group).

### Tissue collection and processing

Rats were given a lethal dose of ketamine/xylazine followed by transcardiac perfusion with ice-cold 0.1 M phosphate buffered saline (PBS) after which brains were quickly removed. Cerebral hemispheres were separated in the midline. The right side was immersion fixed in 3% buffered paraformaldehyde and after several days in fixative the cerebral hemisphere was cut into four coronal slices and embedded in paraffin. The left side was dissected into frontal and parietal dorsal cerebrum samples (which include cerebral cortex and periventricular white matter dorsal to the lateral ventricle, but no striatum) that were frozen in liquid nitrogen and stored at -80°C. It should be noted that the fixed brain and tissue homogenates from the 1 week, 3 week, 7 week, and 10 month rats have also been used for other previously published experiments [27-29]. Our approach to tissue handling has been consistent.

### Western blotting

Frozen frontal and parietal cerebrum samples were pooled together, weighed and homogenized in three times volume of ice-cold RIPA buffer containing 1% deoxycholic acid, (D5670, Sigma-Aldrich, Oakville ON, Canada), 0.1% sodium dodecylsulfate, (BP-166, Thermo Fisher Scientific, Waltham MA, USA), 1% Triton ×-100, (T9284 Sigma), in 0.1 M PBS pH 7.4 containing 0.1 mg/ml phenylmethylsulfonyl fluoride, (PMSF, P7626 Sigma) and 3% (v/v) aprotinin, (A3428 Sigma). The tubes were allowed to sit on ice for 30 min prior to centrifugation. The supernatant was removed after the first centrifugation at 15,000 ×G for 30 min at 4°C and centrifugation repeated. The second supernatant was aliquoted and stored at -80°C. Total protein assays were performed on each aliquot using the Pierce Micro BCA protein assay kit (Pierce Biotechnology, Rockford IL, USA). For each antibody, equal quantities of protein homogenate samples were run on 6% acrylamide/SDS gels except for dermatan sulfate, which were run on 8% gels. Samples for phosphacan were digested with chondroitinase ABC (C3667 Sigma) [30] prior to running on the gels, along with prestained molecular weight markers (161–0324 Bio-Rad Laboratories, Hercules CA, USA) or biotinylated protein ladder (7727S New England Biolabs, Ipswich MA, USA,). Laminin positive control was from mouse sarcoma (L2020 Sigma). Extracts from each age group were run on a separate minigel in order to compare hydrocephalics with age-matched controls. Gels were electrophoresed for 90 min, equilibrated in transfer buffer containing 20% methanol, and then transferred onto Hybond ECL nitrocellulose membranes (RPN2020D Amersham Biosciences, Pittsburgh PA, USA) at 180 mAmp for 3 h. Pierce Starting Block (37542 Fisher) was used to block all the membranes for 1 h at room temperature, except for laminin, which was blocked with 5% bovine serum albumin fraction V (BP1605 Fisher) in TRIS buffered saline with 0.5% Tween 20 (P1379 Sigma). Primary antibody dilutions were: 1:5000 for phosphacan (MAB5210 Chemicon, Temecula CA, USA) and neurocan (MAB5212 Cedarlane Laboratories Burlington ON, Canada), 1:2000 for decorin (263310 Calbiochem; San Diego CA, USA) and NG2 (AB5320 Chemicon), and 1:250 for laminin (RB-082-A1 Medicorp, Montreal QC, Canada). All antibodies were applied at 4°C overnight in their respective blocking solutions with gentle swirling. Secondary antibodies were biotin-conjugated sheep anti-mouse (515-065-003 Cedarlane) at 1:3000 dilution for phosphacan, neurocan and dermatan sulfate, while NG2 and laminin detection used biotin-conjugated goat anti-rabbit (111-065-144 Cedarlane) at 1:2000 and 1:5000 respectively for 2 h at room temperature. Streptavidin/horseradish peroxidase conjugate was applied at 1:5000 dilution for 1 h at room temperature and ECL Western blotting detection reagents (RPN2106 Amersham) were used to visualize the bands using Hyperfilm ECL (RPN3103K Amersham). Negative controls were run using non-species appropriate secondary antibodies, to reveal non-specific background bands. Band density was quantified with Imaging Research Incorporated MCID software (Cambridge UK). Values were normalized to the total loading bands and compared across control ages using nonparametric Kruskal-Wallis test, while control and hydrocephalic groups were compared using Mann-Whitney U test.

### Microscopy and immunohistochemistry

**Figure 1:**
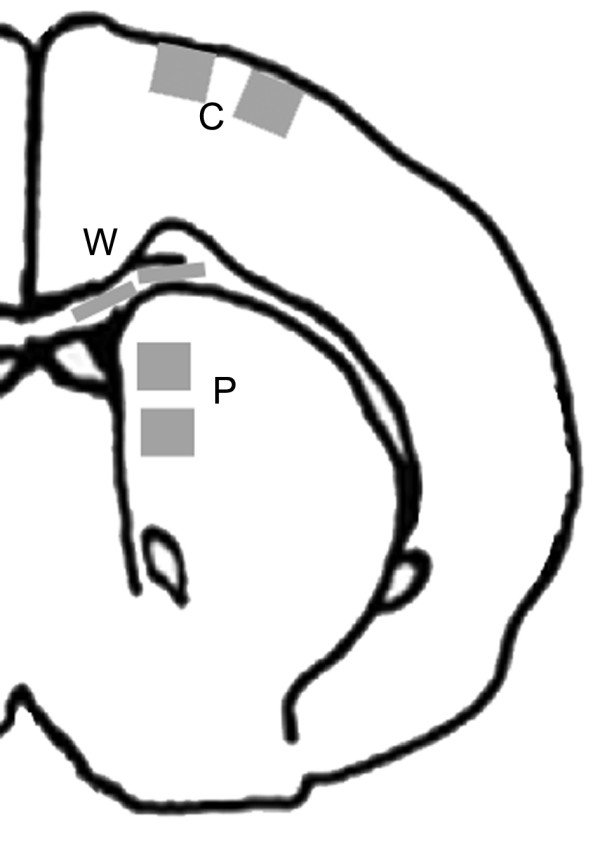
Schematic diagram of rat brain showing shaded areas in which cerebral cortex (C), periventricular white matter (W), and medial caudatoputamen (P) were photographed for image analysis of histochemical staining and immunolabeling.

Paraffin sections (6 μm thick) were stained with hematoxylin and eosin (H&E), periodic acid-Schiff/diastase (PASd; to stain proteoglycans and glycoproteins) and Alcian blue (at pH 2.5; to stain hyaluronic acid and acid mucins including keratan and heparan sulfates). Using standard immunohistochemical methods, various elements of the extracellular matrix were analyzed. Endogenous peroxidases were quenched by treatment with 3% H_2_O_2 _in methanol, and slides were blocked with either 10% normal goat serum or 10% normal sheep serum. We used rabbit polyclonal antibodies against the following proteoglycans and proteins: biglycan (1:2000 dilution; recognizes the core protein; donated by Dr. P. Roughley), NG2 (1:500 dilution; recognizes the core protein; AB5320 Chemicon), laminin (1:500 dilution; RB-082-A1 Medicorp), and mouse monoclonal antibodies against the following proteoglycan core proteins: decorin (1:100 dilution; recognizes core protein of decorin but not biglycan; 263310 Calbiochem, clone 6B6) [30,31], neurocan (1:2000 dilution; recognizes the core protein; MAB5212 Chemicon, clone 650.24) and phosphacan (1:5000 dilution; recognizes the core protein; MAB5210 Chemicon, clone 122.2). Slides were incubated with primary antibodies overnight at 4°C, washed, incubated for 2 h with the appropriate secondary antibody at room temperature, followed by streptavidin/horseradish peroxidase (P0397 DAKO; Glostrup, Denmark) for 30 min, and finally diaminobenzidine (DAB, D5905 Sigma). Four control and 4 hydrocephalic brains were analyzed at each of five time points. Digital photographs of the dorsal frontal cortex, periventricular white matter, and caudatoputamen (two sites each brain; see Figure 1) were obtained at ×200 magnification and converted to grayscale images. The intensity of labeling (relative to normalized background) was assessed quantitatively in sample windows corresponding to the glia limitans, layer 1 of cerebral cortex, layer 2/3 of cerebral cortex, periventricular white matter, and gray matter compartment in the caudatoputamen adjacent to the ventricle using National Institutes of Health (NIH) Image analysis software version 1.62. Data were analyzed using ANOVA with Bonferroni-Dunn test for intergroup comparisons. Values were also compared to the Western blot data by correlation analysis.

### Electron microscopy

Young adult rats underwent kaolin (n = 6) or sham (n = 5) injection, and 2 weeks later T2-weighted MR images of the brain were obtained [13]. The following day the rats were deeply anesthetized with pentobarbital, the calvarium was removed, and immediately a liquid nitrogen-cooled, polished copper block (1 cm × 1 cm contact area) was slammed onto the dorsal surface of the brain [23,24]. The frozen brain was scooped out with the block still applied and both were submerged in liquid nitrogen. Samples of cerebrum were weighed (in pre-weighed aluminum foil packets) then desiccated for 3 days at 100°C. Water content was determined from the difference in the wet-dry weight. For freeze-substitution embedding, fragments from the dorsal cerebrum were removed from the copper block with chilled instruments, placed in 1% osmium tetroxide (OsO_4_) in acetone at -80°C for 2 days, at -30°C for 2 days, then at 4°C for 2 days [23,32]. The fragments were then transferred to acetone/ethylene oxide at room temperature and embedded in epoxy resin. Semithin sections were cut perpendicular to the pial surface and stained with toluidine blue to select pieces that were properly oriented. Ultrathin sections were cut and placed onto copper grids then contrasted with lead citrate. Beginning at the pial surface, a montage series of photographs of the superficial cortex was photographed at 12000× using a JEOL 1010 transmission electron microscope and printed at final magnification of 32400×. Freeze artifact was usually seen at approximately 100 μm depth [9] therefore the extracellular space determinations refer only to layer 1 of the cerebral cortex. The glia limitans was not analyzed. An investigator blinded to the identity of the specimens analyzed two non-overlapping series of 4–6 prints from each rat brain. The extracellular compartment on the photographs was colored and a D64 counting grid enlarged on a transparency was laid over the photograph. The proportion of points (total 1064) over the extracellular compartment was counted using validated stereometric methods [33]. Data were analyzed using unpaired two-tailed Student's t test StatView software, SAS Institute).

## Results

As stated above, the age of the rats refers to the age at time of sacrifice (Table 1).

### Protein detection in cerebrum homogenates

**Figure 2:**
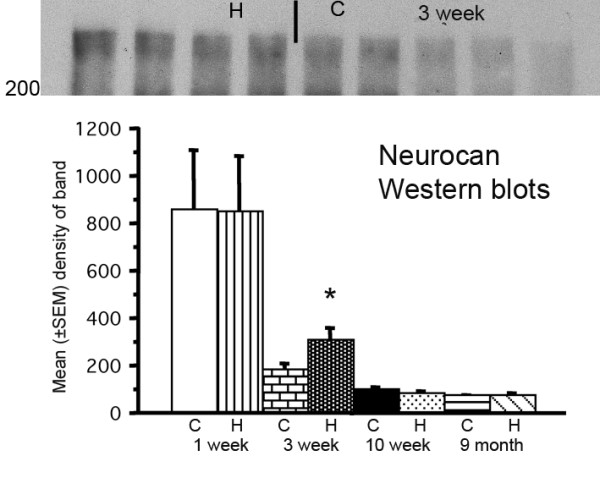
**Western blot of neurocan (molecular size 250–350) (upper panel) detected in cerebrum homogenates of 3-week-old hydrocephalic (H) and control (C) rats. **Quantitative analysis by densitometry (lower panel) showed that only this age was associated with an increase (*p *= 0.0143, Mann-Whitney U test; all groups n = 4–5).

Neurocan was detected at approximately 250–350 kDa [34] after chondroitinase digestion. The bands were prominent at 1 week, less so at 3 weeks, and almost undetectable by 7 weeks. In the 3-week rats (but not at other ages), hydrocephalus was associated with a significantly increased band density (Figure 2). We detected the intact soluble form of phosphacan (350–500 kDa) after chondroitinase digestion, in addition to a lower molecular weight form (180–250 kDa) [35]. Phosphacan declined gradually with maturation (1 week to 10 months) but there were no changes associated with hydrocephalus (not shown). Decorin was detected at about 45 kDa [30]; it was more abundant in the 10 month rat brain than at other ages, but there were no changes associated with hydrocephalus (not shown). NG2, another large proteoglycan 300 kDa in size [20], was readily apparent in animals 1 week to 3 weeks age, but undetectable in the older rats. There were no changes associated with hydrocephalus (not shown). Laminin was detected as a doublet band near 200 kDa and a single band at 400 kDa [36]. It was abundant at 1 and 3 weeks age, decreased significantly by 7 weeks and was nearly undetectable in the oldest rats. There were no changes associated with hydrocephalus (not shown).

### Histological detection of extracellular substances

There was negligible PASd staining of proteoglycans and glycoproteins in 1-week rat brains. In brains from rats 3 weeks and older there was generally an even distribution in the gray matter with slightly more intense staining at the glia limitans and surrounding blood vessels. White matter stained less intensely than gray matter, except in the striatum. In control 1 week and 3 week rat brains the cortex and white matter stained diffusely with Alcian blue. This was negligible in normal rats 7 weeks and older, although scattered neurons in the cortex were surrounded by a narrow but distinct layer, corresponding to the perineuronal nets [37]. For PASd and Alcian blue there was no difference between hydrocephalic and control rats of any age group in any location.

**Figure 3:**
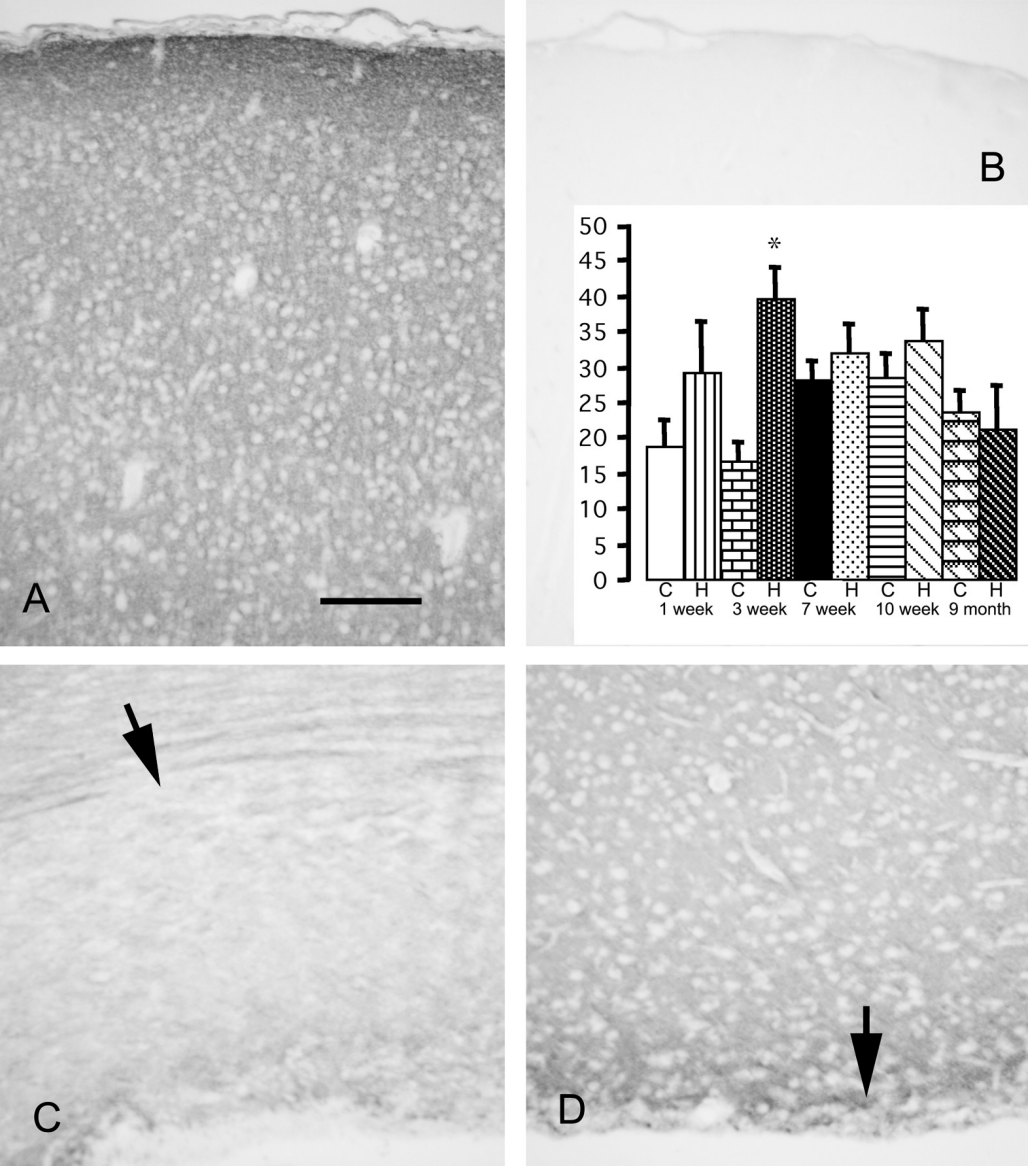
**Photomicrographs showing immunolabeling for neurocan of rat cerebral cortex at 3 weeks of age.** The cerebral cortex of a control brain (A) shows a diffuse extracellular pattern, most prominent in the glia limitans. Hydrocephalic brains appear the same (not shown). Omission of the primary antibody is associated with no labeling (B). Normal white matter (C, arrow) is less intensely labeled. White matter in hydrocephalic brains (D, arrow) is atrophic and the residual tissue is more intensely labeled. Scale bar = 100 μm. Bar graph shows densitometric analysis of immunolabeling (arbitrary units) in white matter of control (C) and hydrocephalic (H) rats. The hydrocephalus-related increase is significant only in the 3-week rats (*p = 0.0007 ANOVA, Bonferroni-Dunn; all groups n = 4). No significant changes were observed in any other location.

**Figure 4:**
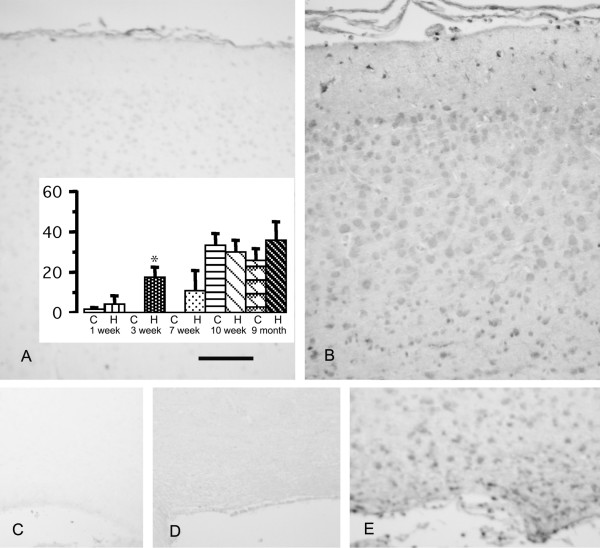
**Photomicrographs showing rat brain immunolabeling for decorin at 3 weeks age.** In control rats, the cerebral cortex (A) and white matter (C) exhibit minimal immunoreactivity. In age matched hydrocephalic rats, perineuronal labeling is increased in the cerebral cortex (B) and periglial labeling is increased in the white matter (E). No labeling is seen in hydrocephalic white matter when the primary antibody is omitted (D). Scale bar = 100 μm. The bar graph shows densitometric analysis of immunolabeling (arbitrary units) in cortex layer 1 of control (C) and hydrocephalic (H) rats. The hydrocephalus-related increase is significant only in the 3-week rats (*p = 0.0003 ANOVA, Bonferroni-Dunn; all groups n = 4). Note that significant changes were also present in the glia limitans.

Neurocan immunoreactivity was present in an extracellular pattern throughout most of the brain tissue, most prominent in the outer cortex and glia limitans. Only white matter labeling was significantly increased in hydrocephalic rats at 3 weeks age (Figure 3). Phosphacan immunoreactivity was similar to that of neurocan, with a prominent extracellular distribution in the cortex and less intense labeling in the white matter. The glia limitans was prominently labeled in rats 12 weeks and older. The frayed periventricular white matter of hydrocephalic rats 1 and 3 weeks age was more intensely labeled than in controls; however this was not statistically significant (not shown). NG2-labeling was not observed in normal brains. In hydrocephalic brains, stellate cells in the white matter were labeled. Decorin, unlike phosphacan and neurocan, exhibited negligible immunoreactivity in control brains at 1 and 3 weeks age but was detectable diffusely in the glia limitans and cortical layer 1 of rats 12 weeks and older. Perineuronal and white matter labeling was more prominent in 1 week and 3 week hydrocephalic rats (Figure 4). In normal rat brains, biglycan immunolabeling appeared very similar to decorin; however no changes were seen in hydrocephalic rats at any age. Laminin was detected mainly at the glia limitans and surrounding blood vessels; there were no changes in hydrocephalic rats. In no age group for any of the substances studied, were changes observed in the caudatoputamen.

### Electron microscopy

**Figure 5:**
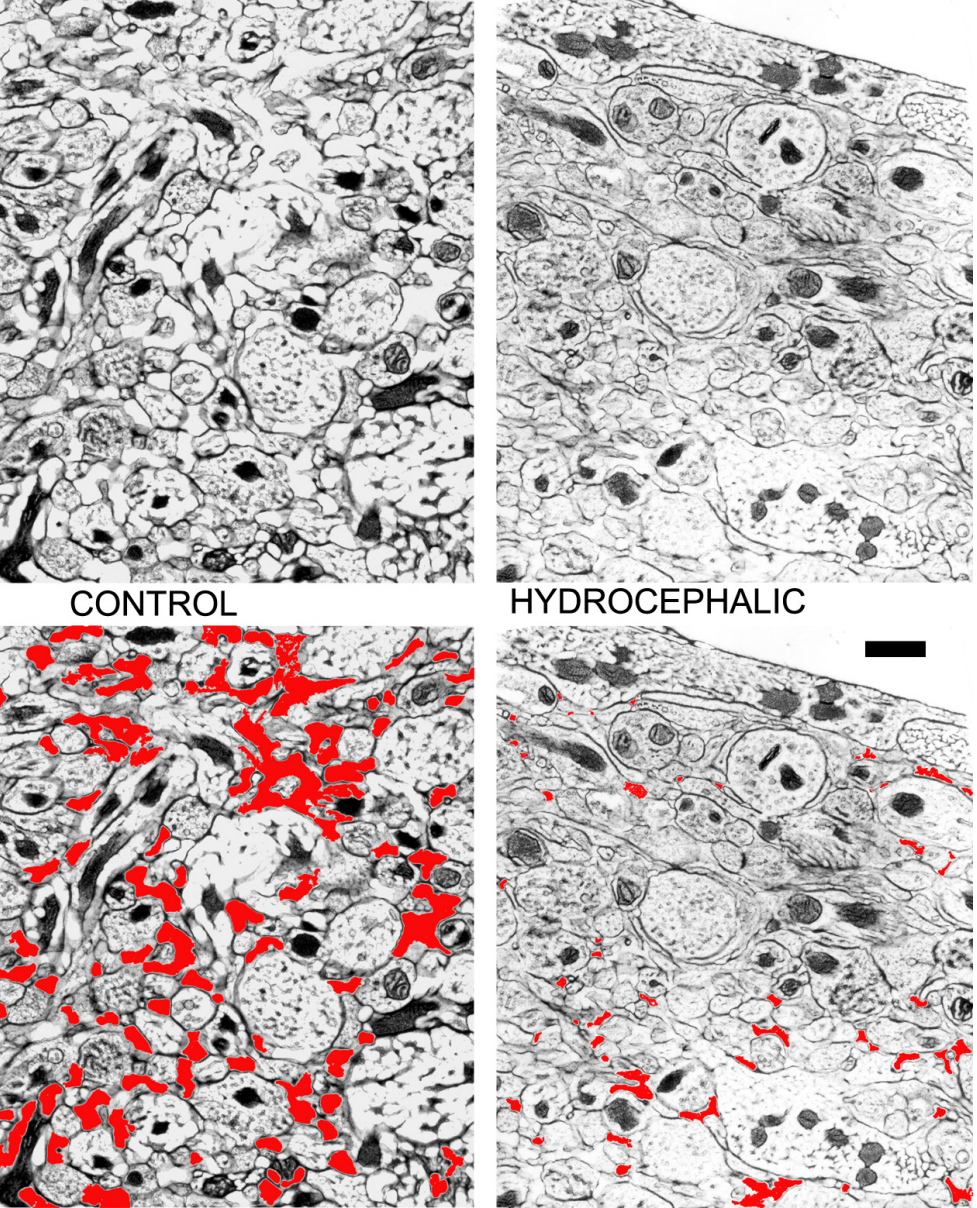
**Sample electron micrographs of cortical layer 1 from control (left) and hydrocephalic (right) 12 week rat brains.** The upper two are the original micrographs and the lower two show the extracellular compartment highlighted in red. Note that the hydrocephalic brain has reduced extracellular space. Scale bar = 0.2 μm

In 12-week-old hydrocephalic rats (duration 2 weeks), water content in the cerebrum (cortex + white matter) was significantly increased (85.0 ± 0.3% hydrocephalic vs. 82.9 ± 0.4% control; *p *= 0.0381). Ultrastructural analysis of freeze substituted samples from layer 1 of the cerebral cortex showed that hydrocephalus was associated with significant compression of the extracellular compartment (relative volume 9.6 ± 1.2% in hydrocephalic vs. 16.5 ± 2.7% in control; *p *= 0.0141) (Figure 5).

## Discussion

A variety of experiments, outlined in the background section, suggest directly or indirectly that the extracellular compartment of the cerebral cortex of hydrocephalic brain is compressed, thereby impairing movement of extracellular solutes. From a functional standpoint, it is important to know if there are permanent compositional changes, because these might impact on the reversibility. Conceivably, the composition might be altered as a consequence of synthesis by reactive cells in the brain. We verified by freeze substitution electron microscopy that the extracellular compartment of cortical layer 1 in hydrocephalic rat brains is indeed compressed. Unfortunately, this method cannot be used to study deeper sites [23,38]. Our previous MR studies using the 12-week rat also indicated that the extracellular compartment of the cortex was compressed, with the ADC reduced in gray matter and increased in white matter [13]. In hydrocephalic cats, the extracellular compartment in the cerebral cortex was reported to be comparable to controls [39]. However, conventional electron microscopy fixation methods are typically associated with cellular swelling and tissue shrinkage that lead to underestimation of the extracellular compartment [26]. Increased water content in the cerebral sample is not contradictory with contracted cortical extracellular compartment. It is likely influenced by the edematous white matter [3], and we cannot exclude the possibility of cortical cell swelling, as has been documented in hydrocephalic cats [40].

In a cDNA microarray screening analysis of hydrocephalic rat brains, we had identified elevations of the mRNA for neuroglycan C precursor in the 7 week and 10 month rats, decreased expression of the mRNA for bamacan in 7 week brains, decreased expression of mRNA for biglycan in 12 week brains, and increased mRNA for biglycan in 10 month brains [41]. Despite this, our screening of extracellular proteoglycans and glycoproteins using quantitative histochemical and antibody methods suggest that compositional changes are minimal, even when hydrocephalus persists for many months. Statistically significant changes limited to neurocan and decorin were seen only in the youngest rats. This does not appear to be dependent on the size or duration of ventriculomegaly because the 7 week and 10 month groups had longer durations of equally severe ventricular enlargement. It should be noted that we have not done a comprehensive assessment of time dependent accumulation from a single starting point. Nevertheless, the data do suggest that 2–3 weeks of hydrocephalus is associated with changes in neonatal-onset, but not juvenile or adult-onset, hydrocephalus. Thus the changes seem to be dependent on the age of the animal. In the youngest rats hydrocephalus began at a developmental stage roughly equivalent to that of a human fetus or premature infant [42]. The relatively greater persistence of chondroitin sulfate proteoglycans (CSPGs) at this age might reflect an attempt by the brain to remain plastic in the situation of ongoing damage [21,43].

Neurocan is a CSPG of the lectican (hyalectan) family. It is secreted primarily by immature neurons and expression decreases after the first postnatal week in rodents [44]. Our findings in control rats are consistent with these data. We observed slight increases in only the white matter of 3-week-old rats with hydrocephalus. The likely source of neurocan here is activated astrocytes [19,45]. Phosphacan, another large CSPG [46], did not change in association with hydrocephalus. NG2, a large transmembrane CSPG that can be cleaved proteolytically to become a secretory molecule [47], was detected around cells in the damaged white matter of hydrocephalic brains. These cells are likely oligodendroglial precursors or macrophages/microglia [48]. Biglycan is a small proteoglycan with chondroitin sulfate and dermatan sulfate side chains. It is upregulated following experimental brain injury [49], but its expression did not change in hydrocephalus. Decorin, a related small leucine-rich proteoglycan exhibited increased immunoreactivity in the superficial cortex and white matter of 3-week hydrocephalic rats. No changes were observed in rats with hydrocephalus that persisted for 9 months.

Our study did not include all brain extracellular substances. We did not assay other proteoglycans of the lectican family (e.g. aggrecan, versican, or brevican), hyaluronan, or tenascin, which all interact in the extracellular compartment [50]. Nor did we examine all of the basement membrane-associated proteins (e.g. collagen type IV, fibronectin, vitronectin, etc.) [51]. Nevertheless, we did sample proteins of various classes and also used nonspecific histochemical staining methods, and found that there were only limited changes in the cortex of the youngest brains or in the white matter where structural damage and reactive changes are known to be prominent in hydrocephalus.

## Conclusion

We conclude that kaolin-induced hydrocephalus in rats is associated with compression of the extracellular compartment in the cerebral cortex. In this experiment we showed this with morphologic techniques for the outer cortical layer of adult rats; others have shown using functional experiments that this is more widespread. There are only minimal changes in the extracellular proteoglycan composition when hydrocephalus begins in the neonatal period, but there are no changes when hydrocephalus begins in the juvenile or adult periods. The volume and tortuosity, which is influenced by the extracellular substances and cell configuration of the extracellular compartment, determine the ability of solutes to diffuse and move by bulk flow [52]. From a functional perspective this is important because impairments in extracellular solute flow associated with hydrocephalus should be largely reversible after correction of the ventriculomegaly.

## List of abbreviations

ADC: Apparent diffusion coefficient, ANOVA: analysis of variance, CSF: cerebrospinal fluid, CSPG: chondroitin sulfate proteoglycan, MR: magnetic resonance, PASd: periodic acid-Schiff/diastase, PBS: phosphate buffered saline.

## Competing interests

The authors declare that they have no competing interests.

## Authors' contributions

MD planned the experiment, did the electron microscopy, analyzed all of the results, and wrote the manuscript. TE performed all of the biochemical and histological assays, including the quantitation. Both authors have read and approved the final version of the manuscript.
